# Successful polio eradication in Uttar Pradesh, India: the pivotal contribution of the Social Mobilization Network, an NGO/UNICEF collaboration

**DOI:** 10.9745/GHSP-D-12-00018

**Published:** 2013-03-21

**Authors:** Ellen A Coates, Silvio Waisbord, Jitendra Awale, Roma Solomon, Rina Dey

**Affiliations:** a Global Consultants, Inc., USA; b The George Washington University, Washington, DC, USA; c CORE Group Polio Project – India, Gurgaon, India

## Abstract

In Uttar Pradesh, India, in response to low routine immunization coverage and ongoing poliovirus circulation, a network of U.S.-based CORE Group member and local nongovernmental organizations partnered with UNICEF, creating the Social Mobilization Network (SMNet). The SMNet's goal was to improve access and reduce family and community resistance to vaccination. The partners trained thousands of mobilizers from high-risk communities to visit households, promote government-run child immunization services, track children's immunization history and encourage vaccination of children missing scheduled vaccinations, and mobilize local opinion leaders. Creative behavior change activities and materials promoted vaccination awareness and safety, household hygiene, sanitation, home diarrheal-disease control, and breastfeeding. Program decision-makers at all levels used household-level data that were aggregated at community and district levels, and senior staff provided rapid feedback and regular capacity-building supervision to field staff. Use of routine project data and targeted research findings offered insights into and informed innovative approaches to overcoming community concerns impacting immunization coverage. While the SMNet worked in the highest-risk, poorly served communities, data suggest that the immunization coverage in SMNet communities was often higher than overall coverage in the district. The partners' organizational and resource differences and complementary technical strengths posed both opportunities and challenges; overcoming them enhanced the partnership's success and contributions.

## Abstract (precis)

Innovative approaches to eradicate polio in hard-to-reach areas included: (1) cadres of trusted community mobilizers who track children's immunization status, (2) responsiveness to people's concerns about immunization, (3) outreach to religious and other local leaders, (4) focus on both individual- and community-level behavioral approaches, and (5) continuous data collection and use.

## THE SETTING FOR POLIO ERADICATION IN INDIA

Polio is a crippling paralytic and potentially fatal disease, spread from person–to-person through poor hygiene and sanitation. Universal immunization against the 3 types of polio with existing safe and effective oral vaccines has been the major strategy for eradicating the poliovirus globally (Box 1).

**Box 1. Four Pillars of the Global Polio Eradication Initiative Strategy**

**1. Routine Immunization**

A major cornerstone of the polio eradication strategy is ensuring that at least 80% of children receive all the recommended routine childhood immunizations, including at least 3 doses of oral polio vaccine, before their first birthday. This would reduce the number of children susceptible to poliovirus, which, in turn, reduces the number of cases, the number of hosts available for the survival of the virus, and the potential for outbreaks.

**2. Supplemental Immunization Activities**

Mass polio immunization campaigns that complement routine immunization programs are intended to interrupt transmission by immunizing every child under the age of 5 with oral polio vaccine annually, regardless of the number of times they have been immunized previously. These campaigns help protect children who are not immunized or only partially protected and boost the immunity of those who are immunized, thereby reducing or eliminating the pool of potential hosts.

These campaigns include National Immunization Days, which are conducted countrywide 2 or 3 times per year, 1 month apart, and subnational Supplemental Immunization Activity campaigns. Although these mass campaigns require careful planning and execution, they are possible because members of the community can be trained easily and quickly to administer the oral polio vaccine safely.

**3. Acute Flaccid Paralysis (AFP) Surveillance**

As many as 90% of people infected with the poliovirus experience very mild or no symptoms.^1^ A single symptomatic case can therefore represent a significant community-wide outbreak. Robust surveillance to detect and investigate every case of polio-like AFP is essential to polio eradication.

**4. Targeted Mop-Up Campaigns**

Low routine immunization coverage, very dense or mobile populations, inadequate sanitation, and poor access to health services exacerbate communities' vulnerability to polio. In focal areas where polio cases have been confirmed within the previous 3 years and circulating virus is confirmed or suspected, mop-up campaigns in which vaccinators go house-to-house to immunize every child under 5 help to stop transmission.

Source: Reference 2.

When the World Health Assembly launched the Global Polio Eradication Initiative (GPEI) in 1988, it was widely acknowledged that India would be one of the most challenging countries for polio eradication, given its enormous and diverse population. In the mid-1990s, an estimated 150,000 polio cases were reported annually in India.^3^ By 2006, Afghanistan, India, Nigeria, and Pakistan were the only remaining polio-endemic countries.^3^

In 1995, the Government of India launched its “Pulse Polio” program for polio eradication, with twice-yearly National Immunization Day (NID) campaigns conducted nationwide and subnational Supplementary Immunization Activity (SIA) campaigns conducted more frequently in selected states.^4^ The government, working with the World Health Organization (WHO) and the U.S. Centers for Disease Control and Prevention (CDC), established the National Polio Surveillance Project (NPSP) to manage polio case detection and reporting.

The U.S. Agency for International Development (USAID) contributed funds to WHO, the United Nations Children's Fund (UNICEF), and Rotary Foundation for surveillance and awareness-raising activities in India. In 1999, USAID provided grant funding for polio activities in Africa and Asia to member organizations of the CORE Group—a global network of international health and development organizations that strengthen local capacity to improve the health and well-being of children and women in developing countries. Recognized for their expertise in working with extremely underserved, high-risk, and vulnerable communities, CORE Group member organizations received funding from USAID and the Bill & Melinda Gates Foundation for the “CORE Group Polio Project” (CGPP) in India.

In 1999, type 2 polio was eradicated worldwide,^1^ leaving only types 1 and 3 poliovirus. That same year, India added a house-to-house polio vaccination effort: after vaccination teams spent 2 days vaccinating children at designated polio vaccination sites, known as booths (Box 2), new teams of outreach vaccinators travelled from house-to-house to locate and vaccinate any missed children.

Of the 3 types of poliovirus, type 2 has been eradicated worldwide and only types 1 and 3 continue to circulate.

**Box 2. Vaccination Campaign Booths**

Temporary booths were established at or near clinics, markets, schools, and places of worship (temples, mosques, churches). Vaccinator teams brought vaccine, cold chain equipment, records, and supplies, and were loaned tables, chairs, and often an awning or tent decorated with flags and posters encouraging families to bring children under 5 to be vaccinated.

Although India reported only 66 polio cases in 2005, it reported 741 in 2009.^5^ This number included 602 cases in Uttar Pradesh (UP) and 117 in Bihar, 2 very vulnerable states with poor sanitation, insufficient infrastructure, and millions of children who require more than the usual 4 oral polio vaccine (OPV) doses for immunity against polio. Research conducted under the auspices of the government, WHO, and other scientific organizations, confirmed that the oral polio vaccine widely used over the last 2 decades is less effective among children in UP.^6^ Ongoing statewide polio campaigns in UP and Bihar succeeded in vaccinating millions of children every 4 to 6 weeks to contain and eliminate the virus. Meanwhile, new polio vaccines were being developed, leading to the introduction in 2005 of monovalent vaccines^7^ (consisting of live, attenuated [weakened] poliovirus strains of either type 1 or type 3 poliovirus) and in 2010 a bivalent vaccine (consisting of live, attenuated poliovirus strains of both type 1 and type 3).^8^ These newer vaccines are more effective against the remaining types of polio.

In 2010, there were only 42 cases of polio in India—with only 10 in UP and 9 in Bihar. The last confirmed case in India occurred on January 13, 2011, in West Bengal.^3^ On February 25, 2012, India was removed from the polio-endemic country list.^3^

In the mid-1990s, an estimated 150,000 polio cases were reported annually in India. By 2012, polio had been eradicated in the country.

Millions of polio field workers in UP and Bihar were crucial to the successful interruption of polio transmission in India. The Government of India and NPSP hired and trained millions of vaccinators and disease surveillance officers. Rotary International members, along with thousands of community-based social mobilizers hired and trained by UNICEF and the CGPP, provided advocacy and social mobilization support for campaigns and routine immunization in high-risk communities using interpersonal communication and mass media. In UP these nongovernmental organizations (NGOs) joined forces to establish and maintain the Social Mobilization Network (SMNet), supported by USAID and the Bill & Melinda Gates Foundation. This paper examines the development, performance, and contributions to polio eradication of the SMNet, including the advantage of individual organizations and networks working together in a consortium focused on a common goal.

## THE SMNET PARTNERSHIP

Initially, Rotary in India received funds from the Rotary Foundation and USAID to support polio campaigns with advocacy efforts at the community level, including with banners, posters, parades, and TV events. As the lead agency for polio communication in India, UNICEF was mandated to coordinate Pulse Polio activities and liaise with the government but had limited field implementation capacity at the start. In its role as one of the spearheading partners of the GPEI, UNICEF also contributed to raising campaign awareness, particularly at the national level, while CGPP partners worked at the community and household level.

Working in the same districts, UNICEF and CGPP became convinced that they could not effectively overcome growing resistance to immunization and ensure universal coverage in high-risk areas by operating independently. Realizing that working separately caused duplication of effort, confusion about roles and responsibilities, and difficulties with linking achievements to either organization's activities, UNICEF, CGPP, and Rotary united within a collaborative framework that allowed them to capitalize on their unique capacities, minimize overlap, reduce friction, share lessons learned, and benefit from a common data collection and monitoring and evaluation approach.

In August 2003, CGPP, Rotary, and UNICEF met with the UP government, which was skeptical at the time regarding NGO involvement in polio eradication. Together, they established the Multiagency Social Mobilization Network known as SMNet, focusing on polio-endemic and high-risk areas in UP. These entities felt that presenting a united front and communicating clear and consistent messages with one voice would make communication with government and other stakeholders more effective. Later, UNICEF introduced SMNet activities in Bihar, where there was no CGPP presence. Although Rotary members continued to support campaigns and promote polio eradication among senior policy makers in India, they were less engaged in the grassroots work supported through the SMNet.^9^

UNICEF conducted increasingly localized social mobilization activities and also brought to SMNet its broad health communication experience, capacity to manage large-scale programs, and ability to raise funds at state levels and then redirect funds to fill other partners' resource gaps. While UNICEF's process for approval of budgetary and programmatic changes could be cumbersome, once approval was received, it could redirect both human and financial resources relatively quickly to emerging high-risk areas as the virus circulated to new communities and as resistance to vaccination services waxed and waned.

CGPP partners systematically engaged with existing international and local NGOs and communities to build trust and address local concerns about polio campaigns, especially in those communities where the government and United Nations (UN) agencies had limited access. Ongoing funding from USAID and the Bill & Melinda Gates Foundation allowed CGPP partners to support immunization and surveillance activities and to support families with paralyzed children. CGPP partners participating in the SMNet included the Adventist Development Relief Agency, Catholic Relief Services (joined in 2003), Project Concern International, and World Vision (left in 2008).

In addition, 10 local community-based NGOs and some Catholic diocesan social service organizations participated in the SMNet. All had established credibility because they had governmental approval to receive funding from abroad. CGPP partnered with these local NGOs to expand or improve their grassroots reach in high-risk communities and to enhance community ownership. Grant funds supported field staff salaries, travel, and capacity-building technical and management oversight.

CGPP's compact structure, long-term community presence, and programmatic and technical flexibility fit the emerging needs of the eradication effort particularly well. The CGPP approach encouraged development of creative, timely interventions and facilitated rapid introduction and assessment of new activities, using community feedback to identify the most effective practices.

Another essential component of the CGPP design was its national secretariats—coordinating units established in each CGPP country to provide leadership, coordinate activities and resource allocation, ensure the quality of CGPP management and technical activities, and serve as a central communication hub connecting field staff, partners, and stakeholders. By design, the secretariats report directly to the independent CGPP headquarters staff in the United States, rather than to a CGPP partner organization. Vested with clear, strong authority, the secretariats are also charged with respecting and maintaining the essentially collaborative nature of the project and the implementing partnership.

The secretariats were required to approve each partner's annual and overall strategic plan and budget and make funding recommendations to the director on behalf of all partners. The partners standardized some aspects of their respective budgets, which was a major contributor to the successful partnership and management of the SMNet. For example, salaries and benefits were consistent across all SMNet field workers, regardless of which CGPP partner paid them. In addition, the partners developed a standardized “cost per census block” to determine activity costs, based on the number of blocks included in each partner's approved catchment area.

The secretariats also represent and speak for the partners at all state, national, and international meetings, voicing their comments, questions, and concerns while allowing them to avoid the costs of participating themselves. The secretariats actively credit the partners and field staff, rather than themselves, with project successes.

SMNet partners use the same vocabulary, messages, and indicators, and they employ similar staffing structures, selection criteria, job descriptions, and benefits. They collaborate with each other and coordinate with local government officials to support routine immunization; campaign planning/microplanning, implementation, and monitoring; and other polio eradication activities.

## THE SMNet FIELD STAFF

**FIGURE 1.**
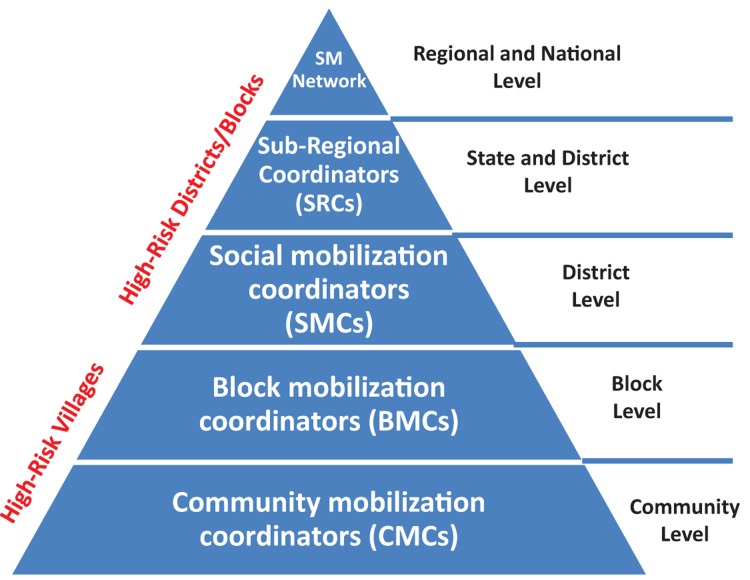
Social Mobilization Network (SMNet) Pyramid Source: Reference 10.

Thousands of social mobilization field workers, trained by CGPP and UNICEF, conducted SMNet activities at different levels (Figure 1). Each cadre of field worker performed different yet interrelated functions in support of polio eradication, routine immunization, and health capacity building.

**FIGURE 2.**
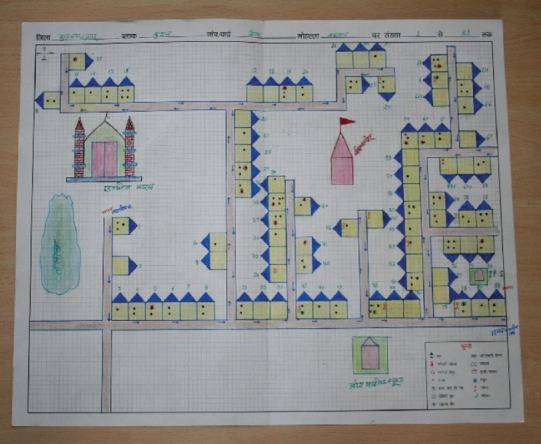
Sample Community Map of Vaccine-Eligible Children

Within the largest cadre, UNICEF managed more than 3,000, and CGPP more than 1,000, **Community Mobilization Coordinators** (CMCs). Predominantly women selected from the high-risk communities, CMCs received small monthly stipends and were responsible for the immunization status of all children under 5 in their assigned blocks, numbering 400–500 households. They maintained detailed maps of their communities (Figure 2) and visited their assigned households at least once each month to promote polio vaccine campaigns. Using specially designed registers, CMCs tracked pregnancies and the routine and polio immunization status of newborns, children under 5, and pregnant women, sending their register data to their supervisors monthly. CMCs visited each household between and during the campaigns to:

- promote child immunization, hygiene, and sanitation
- raise awareness about the importance of routine immunization and polio eradication
- track missed children and ensure that they got vaccinated

During frequent UP-wide campaigns, CMCs organized children's rallies and mosque/temple announcements, helped the vaccinators set up booths, accompanied them to houses of missed children, and assisted in convincing resistant families to have their children vaccinated. At the community level, CMCs participated in routine immunization trainings and service delivery, and they organized mothers' and influencers' meetings, using educational materials and discussions to promote immunization and other positive health-seeking behaviors. SMNet worked to secure the endorsement and active support of influencers, such as political and religious leaders, doctors, athletes, and artists, who were respected and well-known in the community and were consequently able to influence families' decisions and actions.

**Figure:**
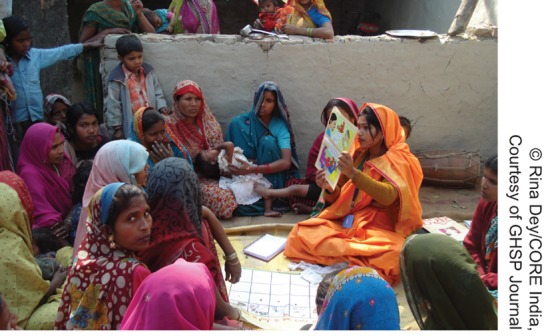
In Uttar Pradesh, India, a community mobilization coordinator promotes immunization and other positive health-seeking behavior at a local mothers’ meeting.

**Block Mobilization Coordinators** (BMCs) trained and supervised the CMCs working within their assigned census blocks and aggregated and analyzed the CMCs' register data. They also worked with local health officials on vaccination campaign microplans, monitored routine immunization sessions, trained NPSP vaccinators on interpersonal communication techniques, and organized health camps and children's rallies. They provided monthly reports to their supervisors. During polio campaigns, BMCs monitored vaccination booths and house-to-house activities supported by CMCs and provided feedback.

The SMNet **District Mobilization Coordinators** (DMCs) trained the BMCs, developed communication plans, and monitored routine immunization sessions between campaigns. During campaigns, they monitored the vaccination booths and house-to-house visits, provided feedback, and updated campaign immunization records. They also collected, aggregated, and analyzed the BMCs' monthly data and compiled district-level assessment reports for the Sub-Regional Coordinators (SRCs) who, with the CGPP Secretariat and state-level UNICEF staff, reviewed and analyzed data from the larger SMNet perspective.

## PROGRAMMATIC CHALLENGES AND INNOVATIVE SMNet SOLUTIONS

The government's Universal Immunization Program, launched in 1985, and the Pulse Polio Program called for immunizing *every* child under age 5 with at least 3 doses of OPV.^11-12^ Achieving that in UP alone meant that vaccinators had to reach more than 40 million children under the age of 5 in every campaign (Box 3). Pockets of children that had not yet been reached within UP's enormous, highly mobile population posed a significant threat to achieving this objective. Nomadic families moved frequently within UP and across the Nepal border. Cultural and language barriers meant children from disenfranchised families, such as bricklayers, slum dwellers, non-Hindi-speaking Muslims, and Scheduled Castes/Tribes (historically disadvantaged families), were often unrecognized, unreachable, and unimmunized.

**Box 3. Sheer Numbers**

Achieving the near universal coverage needed to interrupt transmission required immunizing massive numbers of children. In a 1998 National Immunization Day campaign, 134 million children in India were immunized in a single day.^13^ In 2011, nearly 2.3 million vaccinators immunized roughly 172 million children under 5 during each of 2 National Immunization Days.^3^

Furthermore, as mentioned previously, children in UP often needed more than the recommended 4 doses of OPV to be fully protected. Local suspicion and resistance to vaccinations grew when children were paralyzed by polio despite having received more than 4 vaccine doses. Misinformation and rumors also prompted some community and religious leaders to actively discourage, and even prohibit, participation in vaccination campaigns.

Some community leaders actively discouraged—and even prohibited—participation in vaccination campaigns due to misinformation and rumors.

In addition, the government's intensive focus on polio vaccination—seemingly to the exclusion of providing much-needed basic health and sanitation services in these designated high-risk communities—provoked frustration and campaign fatigue and provided fertile soil for suspicion. Some communities believed the polio campaigns were a continuation of coercive population control measures from the 1970s when the government forced people with 2 or more children to be sterilized. These factors prompted some families to leave home or send their children away during the well-publicized campaigns while others held the polio program hostage, refusing to vaccinate their children until other health services such as sanitation, clean water, and oral rehydration salts/solutions to prevent diarrheal dehydration were made available. In extreme cases, resistant families verbally threatened the campaign workers or threw garbage or boiling water at them.

In response to these challenges, SMNet partners created linkages between the community and government to address local concerns. The SMNet was designed to address various goals, including: addressing parental concerns; understanding reasons for refusing vaccination; creating trust between polio eradication personnel and local residents; tracking missed children including newborns; and identifying missed subpopulations. One common thread in the innovative solutions that the SMNet implemented to achieve these goals was the use of data to inform the approaches. In addition, the SMNet implemented a range of creative communication and social mobilization activities to build community trust and promote vaccination.

### Use of Data to Inform Evidence-Based Approaches

The use of data for program planning and implementation was not new—decision makers used data from NPSP's AFP Surveillance weekly reports to determine the vaccination status of children with AFP, which helped them identify gaps in coverage, prioritize districts and blocks, and direct limited resources strategically. They used coverage and performance data from national and subnational campaigns to assess campaign achievements and plan for the future. As campaign monitoring improved, UP campaign implementers used real-time data in nightly government-led debriefings to support rapid situation analysis and problem-solving.

Despite all this rich data, there were still gaps, particularly at the household level. The SMNet implemented several innovative data collection and use strategies to inform planning, implementation, resource allocation, and message design, including community maps, household registers, a 2-phased vaccination outreach approach, and ongoing community monitoring.

#### Community Maps

The SMNet CMCs began to create community maps (Figure 2) to collect data describing social mobilization activities and child immunization status at the household level. That data were then aggregated monthly at the block, district, regional, and state levels, and then used to identify gaps in implementation.

#### Household Registers

CMCs used CGPP-designed registers to collect and update household-level data during routine monthly household visits and campaigns. These data included pregnancies and vaccination histories (for all antigens in the government's routine immunization schedule) of all children under 5 years of age, enabling CMCs to track the immunization status of individual children and newborns and identify infants and children with missing vaccinations, particularly those consistently missed during the campaigns.

To track households with children needing vaccination, CMCs initially marked houses with children under 5 years of age with either a “P,” indicating that all children under 5 in the household were vaccinated, or an “X,” indicating that the house had at least 1 missed vaccine-eligible child. The primary goal was to reduce resistance and convert “X” houses to “P” houses.

In 2005, SMNet partners and the NPSP realized that they needed more detailed data. The “X” code representing unvaccinated children was expanded to indicate, for example, that the child was away from the village and not expected to return before the end of the campaign (XV), the child was away from the house but would return before the end of the campaign (XH), the child was sick (XS), or the family was openly resistant and refused the vaccine (XR).

This additional information facilitated more targeted responses. For example, when families refused to vaccinate their children claiming they were sick, the CMCs could bring local doctors to their homes to help convince the families that the vaccine would help—not harm—their children. At every SMNet level, this led to an evolving and increasingly sophisticated use of data and understanding of subtle types and causes of resistance, which strengthened the polio eradication effort, built capacity, enhanced quality, and promoted local ownership

**Figure:**
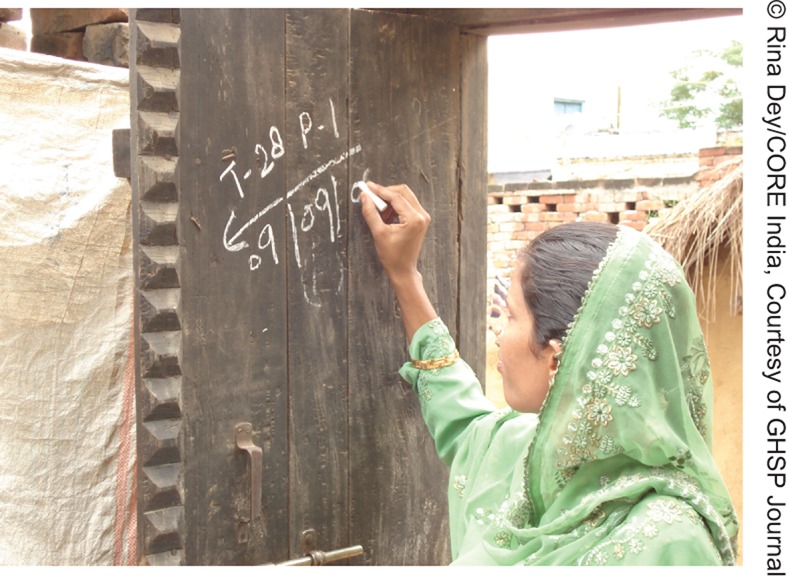
A vaccinator in Uttar Pradesh marks a house with the letter “P,” indicating that all children under 5 in the family were vaccinated.

As described above, CGPP secretariat and UNICEF/UP staff aggregated, analyzed, and used household data from the CMCs for decision-making at each level, and they provided feedback to the field staff. This process instilled valuable skills and a sense of ownership among the SMNet mobilizers at every level.

#### Biphasic House-to-House Vaccination Outreach

At the start, vaccinators were asked to cover very large numbers of children across wide geographic areas, identify missed children, and convince resistant households. Results showed, however, that it was impossible for the 2- or 3-person vaccinator teams to meet all these demands in the time allotted to them.

To remedy this, the government, with CGPP and other stakeholder encouragement and support, introduced A and B vaccination teams with specific and separate tasks. The A team vaccinators and CMCs visited houses first to find out how many children lived in each household, vaccinate as many of the children missed during the booth days as possible, and document any remaining missed children and households (“X”). They were actively encouraged to be honest about the numbers reached and missed. In the past, pressure to report high coverage had encouraged campaign over-reporting. The B team vaccinators and CMCs then visited all remaining “X” houses to work with the families to overcome resistance and vaccinate the missed children. This approach helped to establish more accurate denominators, used vaccinator time more efficiently, and enhanced mobilizers' and vaccinators' interpersonal communications skills. It also improved community mapping and informed training, microplanning, and allocation of teams and vaccine.

The field experience of CMCs and their knowledge of their communities particularly supported the B teams' efforts. Because the households had come to trust the CMCs, they were more open to receiving and discussing their concerns with the B teams.

#### Ongoing Community Monitoring

Over time, the partners began to value data and to invest resources into using household surveys and focus group discussions to assess and strengthen immunization knowledge, attitudes, and practices not only of community members but also of the SMNet field workers and local health workers. For example, a qualitative study showed that some CMCs and local health care providers had resisted vaccinating their own children and that local health care providers were counseling parents to avoid vaccinating children who showed signs of even the most minor illnesses. In response, SMNet partners revised messages and training curricula and adopted more frequent and interactive training approaches to prevent misinformation.

### Communication and Social Mobilization to Promote Vaccination

SMNet partners recognized that relying on traditional widespread distribution of informational materials and outreach activities just before and during campaigns offered limited success given the complexity of the situation, particularly among “resistant” communities. Improved data collection and analysis led to expanded, innovative communication strategies.

#### Interpersonal Communication

Interpersonal communication was at the core of the success of CMCs in building trust and reducing resistance to vaccination. Because vaccinator turnover was high, new vaccinators were often inadequately trained and unsuccessful in addressing families' concerns and fears during household visits. UNICEF and the CGPP secretariat therefore led interpersonal communication training sessions during government vaccinator training programs in SMNet areas.

Community mobilization coordinators played a critical role in reducing community resistance to vaccination.

CMCs also built and maintained supportive local networks of community, religious, and cultural leaders, doctors, teachers, and other respected individuals. With SMNet training and support, these influential opinion leaders were able to respond effectively to local fears and misconceptions.

For example, some **religious leaders** had previously issued *fatwahs* (legal opinions or decrees issued by Muslim scholars) condemning participation in child vaccination. But after they began meeting with CMCs, they adopted SMNet messages, promoted participation in routine and campaign immunization services, and announced upcoming campaigns during worship services and meetings.

Community mobilization coordinators worked with religious leaders and other influential people to promote vaccination campaigns.

**School teachers** played a critical role in organizing SMNet school activities and mobilizing *bulawwa tolies—*children's brigades used to encourage children's participation in polio vaccination campaigns. As trusted sources, **doctors** also helped overcome family resistance to vaccination when their children were sick.

In addition, CMCs earned the trust of **key informants** such as barbers and brick-kiln owners who interacted with migrant workers. These informants helped CMCs locate families of migrant workers who were often missed during campaigns and lost to follow up.

Directly and indirectly, **CMCs were the linchpin in building trust at the community level**. In fact, in recent years the government and other stakeholders have involved SMNet CMCs in ensuring community participation in such activities as a 2009 study comparing the immunogenicity of different doses and methods of administration of polio vaccine among children in Moradabad.^14^ At the request of the government, the CDC, and WHO, CMCs facilitated enrollment of families and collection of multiple blood samples from children. They are credited with achieving higher-than-expected participation and a drop-out rate of less than 7%.

#### Innovative Messages and Materials

Innovative polio eradication messages and materials have been a hallmark of the SMNet, and particularly of CGPP. Continuous communication work across vulnerable communities demanded constant development of new messages and materials that were technically sound and responsive to local data, as well as sensitive to the communities' attitudes and concerns and meaningful for local audiences.

**Figure:**
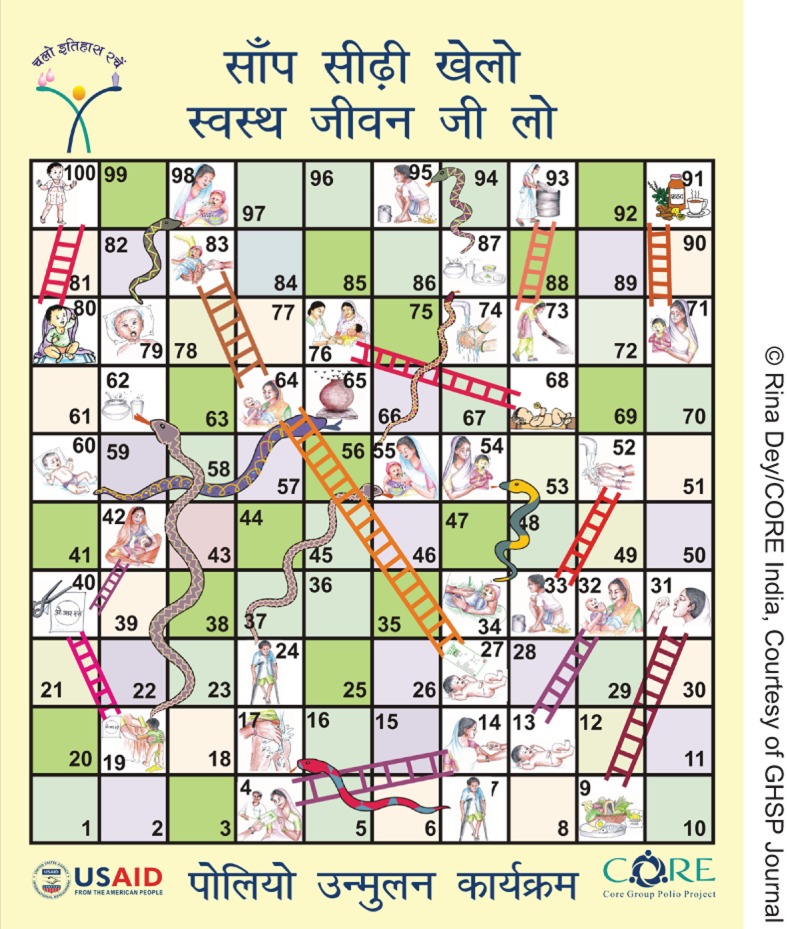
The Core Group Polio Project produced a “Snakes and Ladders” board game with a health theme to encourage children and families to choose positive health behaviors, such as polio vaccination.

Creative behavior change messages and materials have been a hallmark of the SMNet.

**Figure:**
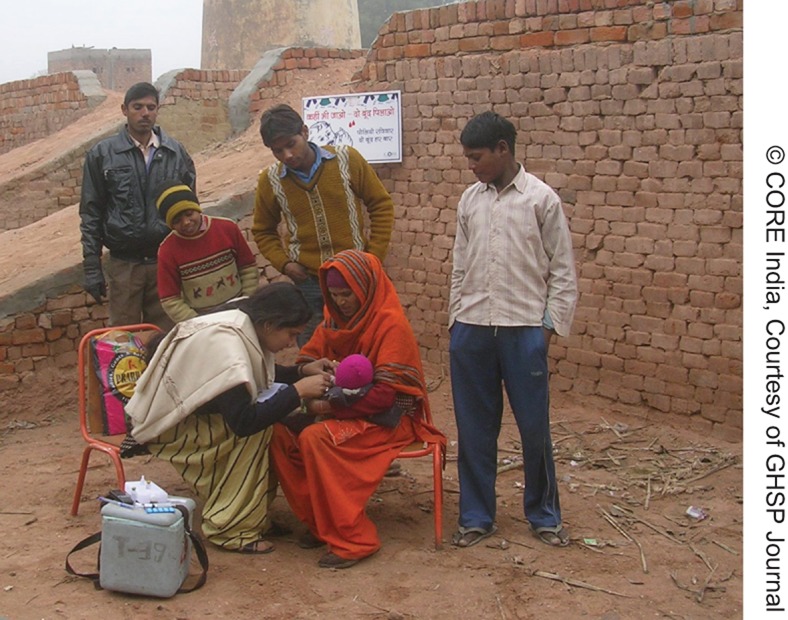
Vaccinators conducted special outreach activities, such as at brick-kiln sites, to reach families of migrant workers in Uttar Pradesh.

Although a broad range of attitudes persist and it is difficult to generalize across communities, in general, local attitudes about OPV shifted from early acceptance, to suspicion and resistance, and then to passive acceptance and signs of growing apathy. Each of these stages required a different communication and social mobilization approach to which the SMNet responded.

Mobilizers from different high-risk, resistant communities met to share challenges and solutions. Insights into complex local attitudes prompted new approaches to overcoming resistance, including messages tailored to specific social and religious groups and their concerns. These were incorporated into creative educational materials, street theater, puppet shows, and other activities promoting immunization and other relevant behaviors, such as handwashing to prevent spreading the poliovirus.

Polio-related materials from the SMNet included:

- Comic books
- Games
- “Science of Polio” video
- PowerPoint presentations
- Picture-based behavior change training modules that CMCs used with mothers during health education sessions
- “Frequently Asked Questions” handout
- Congratulatory cards for new mothers with reminders to protect their babies with the first “zero” polio dose
- Illustrated, scented soap strip packets encouraging handwashing

**Figure:**
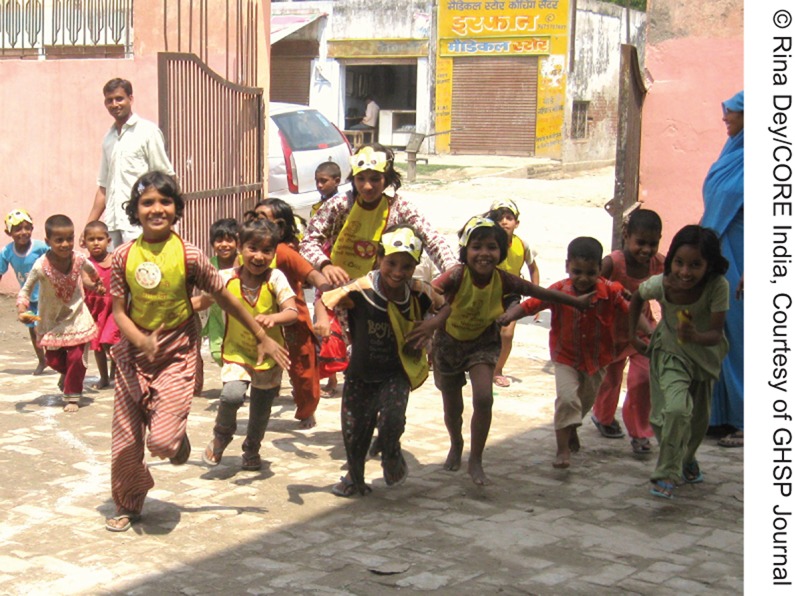
On vaccination booth days, children in Uttar Pradesh encouraged people to vaccinate their children.

Eventually CGPP incorporated other health messages addressing issues such as handwashing and sanitation into their behavior change materials. For example, CGPP designed a game based on the traditional Indian “Snakes and Ladders” game but with game board illustrations promoting immunization and other health-seeking behaviors and discouraging unhealthy practices. Squares illustrated with positive health-seeking behaviors are linked with a ladder; landing on these squares literally gives the player a boost up in life. On the other hand, squares illustrated with unhealthy behaviors are linked with snakes that force players back to positions farther away from the winning square.

A mix of sources informed message development. Research conducted among community members and mobilizers, in particular, provided valuable data. For example, families often did not save the immunization and child health cards distributed by the health system. In response, CGPP produced special bags for families to store the cards and introduced new messages encouraging families to keep their children's cards for 5 years.

The partners also produced specific messages focused on getting children vaccinated *wherever they are* in response to NPSP data showing that 15% of polio cases occurred among migratory populations. CGPP adopted and incorporated key messages from the Global Polio Eradication Initiative and “Facts for Life” messages developed by UNICEF and other UN agencies.

SMNet behavior change communication experts also integrated and field tested local artistic forms and linguistic expressions into key messages to make them relevant to local culture. UNICEF credits CGPP materials with being particularly well developed.

UNICEF and CGPP frequently provided feedback on prototypes and adopted each other's final behavior change and training messages and materials. Typically, materials were tested at both district and community levels. The materials were produced locally and incorporated logos of all partners. Training workshops gave CMCs and BMCs opportunities to discuss and learn to use new materials. An Information-Education-Communication working group focusing on routine immunization materials was established to better systematize and harmonize design and production processes, particularly as the SMNet worked to integrate polio communication materials with routine immunization and other health-seeking behaviors.

#### Child Mobilizers

As early as 2000, CGPP started involving school children to encourage their families to vaccinate their younger siblings. Later, groups of children marched through their communities before campaigns, creating a celebratory atmosphere and encouraging people to protect their children's health by getting them vaccinated. Over time this activity was further refined by the SMNet, and the children's groups became known as *bulawwa tolies* (“calling teams”).

Children’s groups known as *bulawwa tolies* mobilized families to vaccinate their children.

As mentioned above, CMCs worked closely with teachers to engage school children in the polio effort. CGPP recently introduced school programs to educate children about the links between hygiene, sanitation, and health. The program also encourages students to motivate their families to practice good hygiene in their homes, as well as participate in immunization activities.

“Fun Classes” were also recently introduced in more than 500 schools using entertainment formats, coloring books, and class discussions to raise children's awareness about polio, immunization, handwashing, and sanitation. *Kukru-ku* (referring to the “cock-a-doodle-do” morning wake-up call of a rooster) rallies promote use of latrines and discourage open defecation. Children parade through the streets celebrating the value of toilets and putting coveted CGPP-designed nameplates on each house that has its own indoor toilet.

#### Broader Health Initiatives

Faced with long-standing and serious health problems, such as diarrheal diseases, malaria, tuberculosis, malnutrition, and lack of sanitation, communities in UP began questioning why the government regularly provided OPV but seemingly remained inattentive to other urgent challenges. These questions and unmet needs contributed to community suspicion and resistance to polio vaccination.

In the first 2 years of the SMNet, CGPP launched health camps and sanitation drives to address community concerns. Before polio campaigns, government health workers and specialized health providers participated in health camps in public areas outside mosques, schools, and other village sites in high-risk areas. The health camps offered services including antenatal care, routine immunization, and distribution of oral rehydration salts (ORS). Soon the government and other stakeholders began conducting health camps in districts that were not covered by the SMNet while continuing to support CGPP and UNICEF health camps.

The SMNet gained the trust of communities by responding to their demands for broader health initiatives.

Sanitation drive activities ranged from collecting garbage to cleaning city drains and building latrines. Funding for these activities was available only in 2003, but they built local confidence in the health system, and the activities did continue in some areas. Subsequently their value was recognized in the government's “107 high-risk block plan” introduced in 2010 to maximize polio vaccination operations in the 107 high-risk blocks of UP and Bihar.^15-16^

## OPERATIONAL CHALLENGES AND STRENGTHS

The GPEI mandate to “chase and contain” the highly mobile poliovirus posed new challenges to CGPP partners who were used to long-term integrated community health programs. The partners could add polio activities to the child health services that they were providing in several districts. However, they were not working in all communities and did not have capacity to serve, or monitor progress in, all the high-risk communities. The changing epidemiological situation required levels of flexibility and responsiveness beyond the resources of some of the partners, particularly as growing resource constraints and donor fatigue affected the partners.

There also was no precedent to inform development of long-term partnerships between NGOs and the government, and there were concerns about effectively managing and coordinating large numbers of staff. CGPP's size, involving more than 1,000 people serving hundreds of thousands of families in very high-risk communities, was unique among NGO programs. Getting started involved understandable growing pains around coordination and interorganizational trust; this delayed initial development of the SMNet.

Furthermore, while the structure of the SMNet contributed to its success, it also posed challenges. Each partner had to follow its internal organizational requirements, which occasionally caused delays. Waiting to get approval from upper-level offices hindered rapid responses to unexpected needs for the partner directly involved, and it also, in some cases, had ramifications throughout the SMNet. This was especially true when decisions related to time-sensitive issues of community coverage or campaign support.

Staff salary issues also affected the partnership and required joint action and decisions. In particular, UNICEF offered better remuneration than CGPP partners. CGPP and UNICEF initially agreed to pay comparable salaries. For some years, the infusion of fresh funds allowed CGPP to keep salaries reasonably on par with UNICEF's, but it became difficult for CGPP to maintain this agreement over the long term due to fluctuations in funding, resources, and priorities.

The SMNet implemented a number of management approaches to resolve these challenges—ranging from transparent communication between all staff levels and stakeholders and defining clear roles of each partner to promoting a unified identity and creating a supportive work environment.

### Top-Down and Bottom-Up Communication Approaches

Continuous and transparent communication and ongoing data-sharing among all SMNet levels, from grassroots CMCs to high-level policymakers, helped to ensure that all partners became aware of any field challenges without delay and responded rapidly with appropriate policies and resources. This contributed to trust and respect both within the SMNet and the communities. Within the SMNet, this fostered ownership and participatory approaches to identifying and solving problems.

A somewhat similar system developed between the CGPP Secretariat and UNICEF's SMNet representative at the state level and the government, NPSP, USAID, and WHO at state, national, and regional levels. The SMNet's data use and transparency gave the SMNet spokespersons credibility and the communities a powerful voice. Invitations for SMNet representatives from CGPP and UNICEF to speak at higher-level immunization program coordination, policy, and technical meetings increased. Conversely, timely information from the government and donors regarding campaigns, new vaccines, innovative best practices, and emerging strategies was communicated rapidly back to field workers and their communities.

### Clear Roles, Responsibilities, and Teamwork

SMNet partners had to trust each other and prioritize and coordinate common actions and goals. Eventually they drafted the document, “Joint Instructions to the Field,” which clearly articulated each partner's roles and responsibilities at every level. This document helped capitalize on the partners' different strengths and minimized confusion, overlap, and misunderstandings.

Formal meetings with clear structures, agendas, and minutes also facilitated coordination and ensured common and timely understanding of program developments and needs. During typical meetings, the partners reviewed previous decisions, recent performance, state-level campaigns, immunization and surveillance data, and the current status of polio eradication. They also discussed community- and CMC-related issues and logistics planning.

Although CGPP and UNICEF worked in the same districts, they agreed to divide up the coverage areas. At the UP government's suggestion, the partners evaluated each district's needs and partner presence. If a partner working in a high-risk area was unable to take on polio activities, the other partners were generally willing to shift resources and fill gaps. In certain cases, the partners identified and engaged new partners already working in those areas. An NGO Assessment Tool emerged from this activity to identify appropriate and credible local NGO partners in new areas.

The SMNet partners worked together to ensure vaccination coverage in all high-risk areas.

### Flexibility

There was no blueprint to guide the innovative structure or development of the SMNet or how it should function. CGPP, UNICEF, and the local NGOs working in partnership with them created the SMNet iteratively, experimenting with new ideas to address management and technical challenges, learning from field experiences, and adapting to meet expressed needs. This flexibility and the trust that developed over time allowed the SMNet to operate efficiently and effectively.

In addition to organizational flexibility, the partners had an unusual level of technical program flexibility within the context of GPEI and government polio eradication strategies and immunization coverage goals. When CGPP was first established, everyone expected polio would be eradicated within a few years. Funding was awarded based on annual work plans rather than on a longer-term, detailed implementation plan with clear activities, indicators, and objectives that is typical of USAID child health grants. The resulting flexibility was a golden opportunity for experimentation; the project's emphasis on quality data collection, analysis and monitoring, and constant communication with stakeholders allowed the partners to identify and disseminate successful innovations.

### Unified Identity

Over time, the partners worked in increasingly close coordination and cooperation and publicly presented consistent messages with one SMNet voice. They also promoted all materials with the SMNet brand including all partners' logos, regardless of which partner was the original author—an unusual step for organizations that typically compete for funds and use their own unique branding to highlight any achievements that they can claim.

The decision to work jointly to address common problems, make common presentations, and represent the partnership as a unit came with maturation of the SMNet. As visible, genuine collaboration at the central level grew, it fostered trust, cooperation, and partnership at the SRC, BMC, and CMC levels between both organizations. The collaborative spirit also encouraged the partners to respect each other's unique capacities, share their experiments, successes and failures, and adopt each other's innovative activities and materials, all under the umbrella of the SMNet brand.

### “War on Polio” Mentality

Ultimately the SMNet partners began to view the polio eradication effort as a “war” against the virus, adopting the attitude that they would do whatever it took to beat the enemy. This approach to confronting the virus—as a constant emergency requiring rapid responses—helped to energize, mobilize, and unite the field workers. As challenges emerged, rather than trying to shift responsibility or assign blame, the partners worked together to overcome each challenge.

### Supportive Work Environment

When speaking about their experiences with the polio eradication efforts in India, many SMNet staff describe their sense of ownership and pride in their contributions to children's health and polio eradication. They also refer to a family atmosphere that the SMNet fostered, which contributed to deep loyalty and a surprising level of retention given work demands. The field staffs' dedication, commitment, and quality of work far surpassed rational expectations, even for staff in the most impoverished regions of UP.

At the same time, special events held to recognize the contributions of CMCs were important as both frequent campaigns and significant outbreaks continued. In 2006, inspired by similar activities in other immunization and health programs in UP, CGPP and then UNICEF introduced jamborees to boost morale and counter worker fatigue. The day-long events, attended by political and cultural leaders and dignitaries, saluted the field staff's work and gave them the opportunity to socialize, play games, enjoy a catered meal, and show off their talents with short skits and music and dancing displays. CMCs also received certificates and trophies honoring their hard work and contributions to polio eradication and child health in their communities.

### Leadership

SMNet was fortunate in having leaders who fostered and supported these technical and managerial innovations, gave staff ownership and latitude to take risks and experiment, and prioritized transparent communication, feedback, and shared resources. While staff at each level played different roles, the leaders treated and respected all staff equally and encouraged everyone to share their ideas and input.

## SMNet CONTRIBUTIONS TO ERADICATING POLIO IN UP

**FIGURE 3.**
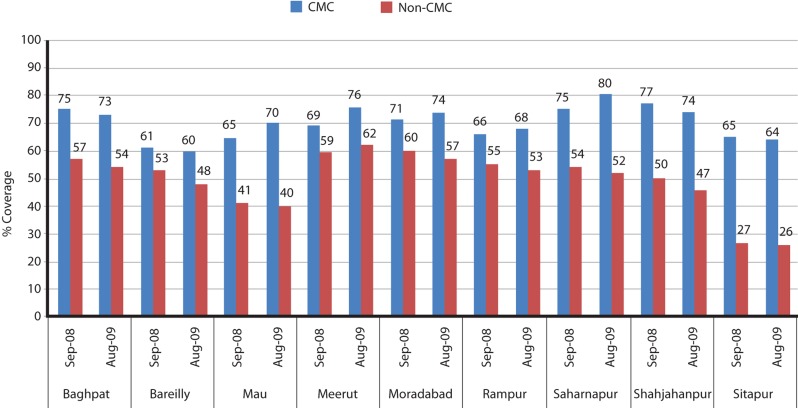
OPV Coverage in Areas With and Without SMNet CMCs, High-Risk Districts of Western Uttar Pradesh,^a^ September 2008 and August 2009 Vaccination Booths ^a^ Muzaffarnagar District is not included because it did not have a vaccination booth in August 2009. Abbreviations: OPV, oral polio vaccine; SMNet, Social Mobilization Network; CMCs, Community Mobilization Coordinators.

Analysis of immunization coverage data in India indicates that the SMNet has had an impact on OPV coverage in highly resistant communities. UNICEF and CGPP do not cover entire districts (many have populations of well over 1 million). Rather, in coordination with the government at the local and state level, they take responsibility for the most underserved, high-risk blocks in poorer-performing districts. Therefore, the CMC-assigned communities in which SMNet partners work are typically those with the highest levels of resistance and low immunization coverage. Despite this, immunization coverage data from September 2008 and from 1 year later in August 2009 show that the CMC-assigned areas achieved substantially higher proportions of OPV-vaccinated children at vaccination booths than areas without CMCs (Figure 3).

The polio eradication effort is frequently criticized for possibly emphasizing polio in ways that distract from, and perhaps undermine, overall routine immunization services. However, all SMNet CMCs actively promoted and supported routine immunization and other positive health-seeking behaviors in their assigned households and communities. CMCs tracked all scheduled child vaccinations in their household registers and followed-up on all missed doses.

**FIGURE 4.**
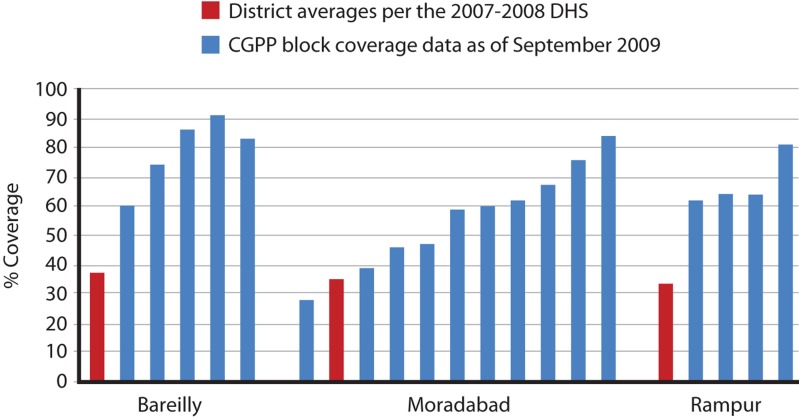
DPT3 Immunization Coverage Among Children Ages 12–23 Months in 3 High-Risk Districts of Western Uttar Pradesh, by District and CGPP blocks Abbreviations: DPT3, 3^rd^ dose of the diphtheria, pertussis, and tetanus vaccine; CGPP, Core Group Polio Project; DHS, Demographic and Health Survey.

The third dose of the diphtheria, pertussis, and tetanus vaccine (DPT3) is generally an accepted proxy for full immunization. Figure 4 compares DPT3 coverage rates among children 12 to 23 months of age in SMNet blocks covered by the CGPP in Bareilly, Moradabad, and Rampur districts with the annual district-wide average for DPT3 coverage for the same age cohort in each of these same districts. The government and WHO recognized these 3 UP districts as very high GPEI priorities. Although the CGPP CMCs work in the districts' highest-risk areas and often serve communities with the poorest access to government services, all but 1 CGPP block had higher DPT3 coverage than the district as a whole (Figure 4).

As further evidence of the SMNet's recognized successful approach, when a polio case was identified in West Bengal in 2011, the government and other stakeholders turned to the SMNet, requesting CGPP to support the rapid response, particularly at the household and community level. Following CGPP's engagement, no additional cases were found.

### Plausibility of the SMNet's Contribution to Eradication

It is fair to ask how much of the polio eradication success in India can be attributed to the SMNet, given the contributions of other components, including a new highly effective monovalent vaccine. Historically, epidemiologic data implicates the crucial role that hard-to-reach children in inadequate sanitation environments play in perpetuating epidemics.^17^ SMNet activities focused on 3 of the 4 elements of the GPEI strategy to eradicate polio—routine, supplemental, and mop-up immunization. Data reveal that SMNet engagement resulted in better coverage for polio and other childhood vaccines, despite—or perhaps because—they focused on higher-risk, difficult-to-reach communities (Figures 3 and ^4^).

Moreover, the response to an outbreak in West Bengal using the same approach was largely credited with snuffing out further transmission there. While of course it is not possible to say definitively that the SMNet was crucial for polio eradication in India, it certainly seems quite plausible. Indeed in countries such as Nigeria and Pakistan, where polio eradication remains stubbornly elusive, the major impediment appears to be local attitudes and suspicion toward program efforts by the local population.^18^ Local adaptation of some SMNet approaches might be very helpful in ongoing eradication efforts.

## SYSTEM-WIDE CONTRIBUTIONS OF THE SMNet

Although India is officially polio free, SMNet efforts have strengthened a wide variety of NGOs and mobilized community members who continue to support and promote routine immunization coverage and access to primary health services. In addition, the SMNet helped strengthen local capacity among hundreds of CMCs, the majority of whom are women. Many CMCs describe the experience as life-changing. CMCs have participated in networks with Anganwadi workers (community workers who link families with organized health care services), auxiliary nurse midwives, private practitioners, and local traditional birth attendants to support not only immunization but also other aspects of maternal and child health. They express pride in contributing to winning the “war on polio” and improving child health and say they have gained valuable skills and self confidence. Many returned to school to study information technology, and at least one went to medical school. There is now a critical mass of female mobilizers with health knowledge and communication skills who can support other health efforts.

While campaigns are admittedly costly, and costs of the GPEI have exceeded initial expectations, the return on the investment in terms of lives saved, paralysis averted, and productivity improved will grow every year. Already, an estimated 5 million children worldwide have been saved from crippling, and possibly life-threatening, paralysis due to polio since the global eradication effort began.^19^ Other benefits include strengthened NGO and community-based health promotion/disease prevention programs and activities, as well as a model to support effective collaboration between NGOs, government, and multilateral agencies, such as UNICEF.

## LESSONS LEARNED FROM THE SMNet EXPERIENCE

The SMNet experience offers important practical and conceptual lessons for health communication and social mobilization, as well as for partnerships in global health. Namely, the partners implemented community-based activities grounded in:

1. Sensitivity to community concerns and demands, good-faith efforts to respond within constraints of available resources, and ongoing, transparent, 2-way communication with communities
2. Strategic use of data at every level, from planning and message development to results monitoring
3. Use of the CGPP secretariat model for management, coordination, quality assurance, and timely dissemination of information internally and externally
4. A wide variety of innovations, including the mobilization of local CMCs, use of household registers, engagement of community leaders, use of child motivators and children's parades, introduction of health camps and support of related health interventions, such as sanitation, hygiene education, and maternal health

While polio eradication in India presented an unusual situation, which was conducive to a campaign mentality with resources that allowed for more intensity and innovation than most health programming, we believe that some of these lessons may be applicable not only to other campaign contexts but also to broader health programming.
